# Depression and correlates of suicidal thoughts among mothers who are female sex workers in three low- and middle-income countries in Sub-Saharan Africa: a cross-sectional application of the Edinburgh postnatal depression scale

**DOI:** 10.3389/fpubh.2025.1656025

**Published:** 2025-10-30

**Authors:** Wendy L. Macias-Konstantopoulos, Regan A. Moss, Brian Willis

**Affiliations:** ^1^Center for Social Justice and Health Equity, Department of Emergency Medicine, Massachusetts General Hospital, Harvard Medical School, Boston, MA, United States; ^2^Global Health Promise, Portland, OR, United States; ^3^Department of Social, Behavioral, Population Health Sciences, Weatherhead School of Public Health, Tulane University, New Orleans, LA, United States

**Keywords:** hard-to-reach populations, social vulnerabilities, psychosocial stressors, mental health, depression screening, suicide risk, thematic analysis, female sex worker

## Abstract

**Introduction:**

Depression is prevalent among female sex workers (FSW) in low- and middle-income countries (LMIC) and poses an elevated risk of suicide. The current study employs the Edinburgh Postnatal Depression Scale (EPDS) as a means of estimating “probable depression” and exploring correlates of suicidal thoughts in Kenyan, Nigerian, and Congolese mothers who are FSW (MFSW).

**Methods:**

This cross-sectional study surveyed a convenience sample of MFSW from eight cities in Kenya, Nigeria, and the Democratic Republic of the Congo (DRC). A sociodemographic questionnaire and the EPDS screener were administered. An EPDS cut-off score of 13 was used to define *probable* depression (score ≥14, 95% specificity). Descriptive statistics and thematic analysis of qualitative data volunteered by participants during the administration of the EPDS are reported.

**Results:**

Among 831 MFSW included, the mean age was 27.7 years and the majority had primary childcare responsibilities for up to 4 children. The highest mean EPDS scores were 24.6 in DRC and 23.3 among MFSW aged 18–24 years, respectively. The pooled mean EPDS score was 22.3 and the sample-based prevalence of *probable* depression was 96.5%. Correlates of suicidal thoughts based on participant responses pointed to themes of desperation, deprivation, isolation, marginalization, criminalization, and traumatization in daily life experiences with prominent correlates including feelings of despair, chronic food insecurity, financial insecurity, lack of work, unsafe living conditions, and traumatic experiences.

**Discussion:**

In the absence of more conventional scales for depression diagnosis and severity, the brief, low-cost, and low-resource EPDS screening tool may be useful among MFSW populations and reasonable for depression detection and referral to diagnosis and treatment. Contextual narratives volunteered in response to the EPDS self-harm question offer insights into the correlates of suicidal thoughts. This study highlights the urgent need for targeted interventions, including mental health services, social programs, policies, and legal protections, to address the mental health of MFSW in LMIC.

## Introduction

Depression is among the most common affective disorders experienced during pregnancy and up to one year postpartum with the potential for adverse outcomes in the mother, child, initiation of breastfeeding, and maternal–infant bond (1–3). Perinatal depression (PND) has been associated with higher rates of pregnancy complications, including pre-eclampsia, preterm birth, cesarean section, and low birth weight (LBW) (4–7), as well as neurodevelopmental delays, learning disorders, and socioemotional and behavioral difficulties for the child (8–12).

Globally, the prevalence of perinatal depression varies by country with notable differences between high-income countries (HICs) and low- and middle-income countries (LMICs). According to the World Health Organization (WHO), PND impacts approximately 1 in 10 women in HICs and 1 in 5 women in LMICs (13). A systematic review and meta-analysis of studies published through 2017, found that antenatal depression was higher in low-income countries (LICs) with a pooled prevalence of 35% as compared to the pooled prevalence of 22.7% in middle-income countries (MICs) (5). Overall, the mean prevalence of PND has been estimated at 26.3% of women globally, with antenatal and postpartum depression prevalence rates of 28.5 and 27.6%, respectively (14). Moreover, while the mean prevalence of PND is estimated at 24.5% in general populations, among vulnerable populations, 1 in 3 perinatal women live with depression reflecting a markedly higher mean prevalence of 32.5% (14). Included among the populations considered vulnerable are immigrant, incarcerated, and HIV-positive women, as well as those with substance use disorder and women who have experienced military conflict and natural disasters (14, 15). In the general population, research has established a strong link between PND and all forms of intimate partner violence (IPV) (15). Additional predictors of PND include any form of abuse or violence experienced across the lifespan, pregnancy-related maternal IPV, unplanned pregnancy, lack of social support, unemployment, and pre-existing mental illness (14–16). Additionally, a recent meta-analysis reported a PND prevalence of 29.5% among women in China, Iran, Pakistan, and Turkey who experienced the COVID-19 pandemic during the perinatal period raising questions about the specific pandemic-related stressors (e.g., isolation, increased IPV, limited access to services, etc.) associated with PND risk (15).

Due to the hazardous socioecological environments in which they live and work, female sex workers (FSWs) experience a heavy burden of wide-ranging psychosocial stressors including IPV; physical, sexual, psychological, emotional, and economic violence; social stigmatization; polyvictimization; criminalization; poverty; food insecurity; housing instability; illicit drug use; sexually transmitted infections including HIV; and limited access to health and social services (17–22). Research has shown that together, these stressors create an increased risk for mental health disorders and disease and, ultimately, exacerbate the social vulnerability of FSWs (23). In the LMICs of India, Uganda, and Nepal, the prevalence of depression among FSWs has been estimated at 39, 48, and 82%, respectively (24–26). Among a cohort of 3000 FSWs in South Africa, the prevalence of depression was 52.7%, compared to 23% prevalence among the general population of South African women, and was associated with stigma, food insecurity, IPV, and HIV-positivity (27). A systematic review and meta-analysis found major depression across 41 FSW studies ranged from 3.3 to 100% prevalence, with one subgroup, FSWs living with HIV, at markedly higher risk for depression (28).

Depressive disorders constitute a major risk factor for suicidal ideation, self-injurious behavior, suicide attempt, and suicide death (29). A recent systematic review and meta-analysis found the pooled prevalence rates for depression and recent suicidal ideation to be 41.8 and 22.8%, respectively, among nearly 25,000 FSWs in LMICs (30). A study conducted in 2019 found that suicide comprised 13.6% of all FSW deaths identified across eight LMICs (31). Among these suicides, 38% were non-perinatal, while 62% of were perinatal with more than half of these (57.8%) carried out in the prenatal period by pregnant FSWs through means such as poisoning, hanging, overdosing, self-stabbing, and self-immolation (32). In the general population, however, a recent meta-analysis including over 6 million perinatal women estimated the pooled worldwide prevalence of *suicide attempts* in the prenatal period was 0.68%, a stark contrast from the prevalence of *completed suicides* reported among marginalized FSWs (33). Yet despite these new insights into alarming rates of perinatal suicide, the prevalence of PND among pregnant and postpartum FSWs in LMICs remains understudied and poorly addressed.

Although originally developed as a screening tool for detecting postnatal depression, the Edinburgh Postnatal Depression Scale (EPDS) has also been validated for the detection of prenatal depression in pregnant women (with cut-off scores of 10 in the first trimester and 11 in the second and third trimester offering optimal sensitivity, specificity, and positive predictive value) as well as for depression in non-perinatal populations (referred to as the Edinburgh Depression Scale, EDS, in non-perinatal contexts) (34–40). Moreover, validation studies for use the tool in perinatal African populations found that a cut-off score of 9 offers optimized sensitivity (94%) and specificity (77%) for depression (41), a reliability profile that approaches diagnostic capabilities. A brief, low-cost alternative to lengthy diagnostic interviews, the EPDS is the most used screening tool in perinatal care and is particularly suitable for identifying probable depression in resource-constrained settings like sub-Saharan Africa (15, 42, 43).

In the current study, the EPDS screener was employed to determine the sample-based prevalence of *probable* depression among a population of stigmatized mothers with poor access to formal health care services. Focused on mothers who are female sex worker (MFSW) in the sub-Saharan countries of Kenya and Nigeria (lower middle-income) (44) and DRC (low-income) (44), this study expands on the results of previous studies conducted across eight LMICs that found disproportionately higher suicide deaths in the three countries selected for this study (31, 32). Moreover, this study explores the contextual narratives of MFSW participants to shed light on socioecological factors associated with suicidal thoughts in this hard-to-reach (HtR) marginalized population of women.

## Methods

### Study design and setting

A community-based, cross-sectional examination of the maternal, mental, and nutritional health of FSWs was conducted across three LMICs in sub-Saharan Africa: in Kenya and Nigeria from February to April 2022 and in the DRC from January to February 2023. The current analysis is restricted to the mental health component of the larger study. Countries included in this study met the following criteria: a large FSW population, a high maternal mortality rate, a high HIV infection rate among FSWs, and access to partnerships with local sex worker organizations (SWOs) and non-governmental organizations (NGOs) working with FSW populations. Focused on the three countries with the highest death and suicide counts among FSW in a 2019 study (31, 32), eight cities in sub-Saharan Africa were identified for recruitment: Nairobi, Mombasa, and Kisumu in Kenya; Lagos, Calabar, and Abuja in Nigeria; and Bukavu and Kinshasa in DRC.

### Study participants and recruitment

Convenience sampling was used to recruit roughly equal-sized samples of MFSW per city (Table 1). A sample size of approximately 100 participants from each of the eight cities was determined to be adequate for representation of local MFSW communities given the limited time and resources available for field investigation. Potential participants were identified by SWO partners in Kenya and Nigeria, and by local NGOs providing HIV services to FSWs in the DRC. Study eligibility was determined using the following inclusion criteria: age ≥ 18 years, biological mother to at least one child aged ≤ 5 years, engaged in sex work full time in the past 3 years, and socially interactive with other mothers within the FSW community. No information was recorded about FSW evaluated and found to be ineligible or about MFSW offered enrollment who declined to participate. Study enrollment included informed consent during which participants were reminded of the voluntary nature of the study and their right to withdraw from the study at any point in time. For safety and to accommodate different literacy levels, enrolled MFSW consented with an “X” on the informed consent form. Participants were compensated with two meals and the cash equivalent to $16 to 20 USD, as recommended by local partners.

**Table 1:** Field interviews.

Country	City	Participants (*N =* 853)	Interview dates	
Kenya	Nairobi	103	February 2022	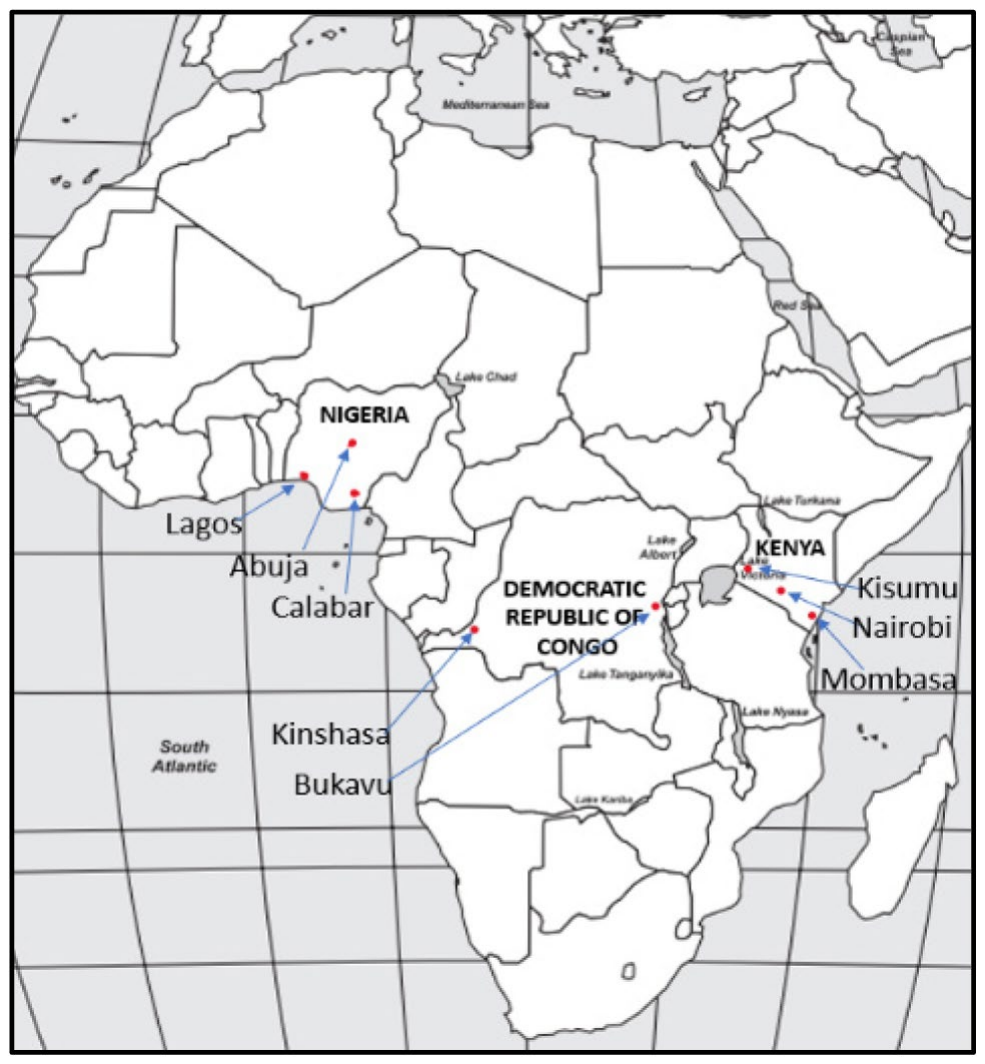
	Mombasa	99	February to March 2022	
	Kisumu	100	March 2022	
Nigeria	Lagos	100	March 2022	
	Calabar	101	April 2022	
	Abuja	100	April 2022	
DRC	Bukavu	100	January to February 2023	
	Kinshasa	150	February 2023	

### Data collection

Data collection was conducted through private face-to-face interviews with participants. Interviews took place in safe and convenient locations as determined by local partners. MFSW participants completed a sociodemographic questionnaire (including age, number of dependent children in the household, perinatal status, HIV status, birth weight of the most recent live birth in the previous 5 years) and the EPDS screening tool. Due to low literacy levels, the EPDS was administered verbally to each participant and her responses were recorded on individual paper forms by trained field staff. The same interviewer administered the EPDS to all participants in the same city.

### Measurement instrument

The EPDS is a 10-item validated tool for the detection of perinatal and non-perinatal depression (34–43). Each item is scored zero to three, with higher scores indicating more serious symptoms. In Kenya and Nigeria, the English language version of the EPDS was used and translated by local partner staff while the validated French version was used in the DRC (45–47). A recent meta-analysis determined that a cut-off score of 11 maximizes the EPDS’s sensitivity and specificity for depression, however, higher cut-off scores are known to yield even greater specificity (42). For this study, the cut-off score of 13 from the standard reference scores (< 8 unlikely, 9–11 possible, 12–13 highly possible, and ≥ 14 probable) (41) was applied and a positive screen was set at ≥ 14 as this carries a specificity of 95% (42). Additionally, item 10 of the EPDS queries the occurrence and frequency of suicidal thoughts in the past 7 days. Specifically, EPDS item-10 states, “*The thought of harming myself has occurred to me*” and subsequently prompts participants to mark one of four options: 0 (*never*), 1 (*hardly ever*), 2 (*sometimes*), and 3 (*yes, quite often*). All participants who scored a 1 or higher on item-10 were referred for counseling, or if mental health services were not available, they were referred to local partner staff with counseling experience.

### Data entry and analysis

Interviewers administered the Edinburg Postnatal Depression Scale and recorded participant responses on paper forms which were subsequently reviewed for completeness by the lead investigator (BW) and another team member. Participant responses to each of the 10 EPDS questions were scored after the interview was completed (35, 48) as they were entered into an Excel database for curation and analysis. Data were cleaned independently by data analysts and compared by two research team members (WLM, RM) for accuracy. EPDS screeners with one or more missing responses were not scored and were excluded from the analysis. Descriptive statistics were performed using Statistical Package for Social Sciences (SPSS, v.29) and XLMiner Analysis ToolPak (49, 50). In addition, any spontaneous explanations or comments volunteered by participants as context for their responses to EPDS item-10 were recorded and a thematic analysis was conducted. The code book was developed using an inductive approach as text data were interpreted for meaning and context, and the emerging themes and concepts were identified. The text data were coded by two independent coders (WLM, RM), and the frequency of recurring themes and concepts (i.e., codes) was quantified. The two coders subsequently met to review, discuss, and reconcile differences. There was inter-rater agreement for 117 of the 125 comments available for thematic analysis, consistent with 93.6% inter-rater reliability. Coding discrepancies discovered were due to 8 comments that could be assigned to multiple codes but were off by a single code between the two independent coders.

### Ethics approval

The study protocol (IRB ID: STUDY0002296) was reviewed and approved by the Institutional Review Board of Oregon Health and Sciences University (Portland, Oregon, USA) and the Ethics Review Committees in each of the three countries: Kenyatta University Centre for Research Ethics and Safety (Nairobi, Kenya), Nigerian Institute of Medical Research (Lagos, Nigeria), and University of Kinshasa School of Public Health (Kinshasa, DRC). All participants gave their written informed consent, which was formally recorded and witnessed by the lead investigator (BW). Our ethical guidelines, standards, and disclosures meet or exceed those required under the Declaration of Helsinki.

### Role of the funding source

The funders played no role in study design, data collection or analysis, results interpretation, and manuscript preparation.

## Results

### Participant characteristics

A total of 853 MFSW participated (302 Kenya, 301 Nigeria, 250 DRC) in the study (Table 2). Mean age of participants was 27.7 with a range of 18 to 52 years. Of those for whom the perinatal status is known (*N =* 682), 41.1% (*n =* 280) were perinatal and 58.9% (*n =* 402) were non-perinatal. While the majority of participants reported a negative HIV status (*n =* 421, 82.5%), 89 MFSW (17.5%) reported HIV positivity. Among participants who could report the birth weight of their most recent pregnancy (*n =* 186), 55 (29.6%) met the definition for LBW.

**Table 2:** Participant characteristics by country.

	Kenya	Nigeria	DRC	Total
Age in years, mean (mode) (*N =* 766)	30.2 (24)	28.3 (25)	24.0 (25)	27.7
No. children in household, *n* (%) (*N =* 853)				
0	1 (0.3)	54 (17.9)	20 (8.0)	75 (8.8)
1–2	155 (51.3)	128 (42.5)	77 (30.8)	360 (42.2)
3–4	124 (41.1)	81 (26.9)	69 (27.6)	274 (32.1)
5–6	20 (6.6)	33 (11.0)	57 (22.8)	110 (12.9)
>6	2 (0.7)	5 (1.7)	27 (10.8)	34 (4.0)
Perinatal status, *n* (%) (*N =* 682)				
Pregnant	41 (17.0)	34 (14.4)	25 (12.6)	100 (14.7)
Postpartum	66 (27.3)	68 (28.9)	46 (23.2)	180 (26.4)
Non-perinatal	132 (54.7)	143 (60.8)	127 (64.2)	402 (58.9)
HIV status, *n* (%) (*N =* 510)				
Positive	51 (18.7)	25 (9.7)	13.0 (10.0)	89 (17.5)
Negative	222 (81.3)	233 (90.3)	117 (90.0)	421 (82.5)
Low birth weight^a^ (*N =* 186)				
Yes	25 (8.9)	6 (8.8)	32 (17.0)	55 (29.6)
No	255 (91.1)	62 (91.2)	156 (83.0)	131 (70.4)

^a^Low birth weight: defined as < 2.5 kg at birth.

### EPDS scores

Of the original sample of 853, the responses from 22 participants (2.6%) were excluded from this analysis due to incomplete EPDS screeners (i.e., participants “declined to answer” or responses missing for one or more questions) and the results of 831 screeners with 100% completeness were included (Table 3). Overall, the average EPDS score was 22.3 with a range of 5 to 30, median of 23, and mode of 24. The mean EPDS score in Kenya (20.16) was slightly lower than the mean scores in Nigeria (22.55) and much lower than in the DRC (24.64). Across three age groups, the mean EPDS score for MFSW aged 30 or older (21.69) was lower than for those aged 18–24 years (23.24) and 25–29 years (22.1). Participants with either no childrearing responsibilities or with >6 child dependents in the household exhibited higher mean EPDS scores, 25.53 and 24.14, respectively, compared to women with 1 to 6 children in the household. Similarly, women reporting a LBW in their most recent pregnancy scored slightly higher on the EPDS (mean 23.5) than their counterparts. Although additional differences in the mean EPDS scores may be expected based on other factors associated with depression, in this sample, no major apparent differences were noted based on perinatal status or HIV status.

**Table 3:** EPDS scores and EPDS item-10 scores by country and known correlates of depression.

	Mean EPDS score	EPDS item-10 scores *n* (%)				Mean item #10 score
		Never (score 0)	Hardly ever (score 1)	Sometimes (score 2)	Yes, quite often (score 3)	
Country						
Kenya (*n =* 294)	20.16	6 (2.48)	4 (1.65)	58 (23.77)	174 (72.00)	1.56
Nigeria (*n =* 295)	22.55	80 (27.21)	15 (5.10)	152 (51.36)	47 (15.98)	1.88
DRC (*n =* 242)	24.64	34 (11.53)	7 (2.37)	212 (71.86)	42 (14.24)	2.65
Age, years						
18–24 (*n =* 271)	23.24	29 (10.66)	4 (1.47)	120 (43.75)	118 (43.38)	2.21
25–29 (*n =* 269)	22.10	31 (11.52)	11 (4.10)	142 (52.42)	85 (31.60)	2.05
30 and over (*n =* 226)	21.49	50 (22.12)	8 (3.54)	128 (56.6)	40 (17.70)	1.70
No. children in household						
0 (*n =* 75)	25.53	10 (8.20)	2 (7.41)	46 (10.72)	17 (6.20)	2.68
1–2 (*n =* 360)	21.69	59 (48.36)	11 (40.74)	195 (45.46)	95 (34.67)	1.91
3–4 (*n =* 273)	21.88	38 (31.15)	13 (48.15)	135 (31.47)	87 (31.75)	1.99
5–6 (*n =* 110)	23.51	13 (10.66)	0 (0)	43 (10.02)	54 (19.71)	2.25
>6 (*n =* 734)	24.14	2 (1.64)	1 (3.70)	10 (2.33)	21 (7.66)	2.47
Perinatal status						
Pregnant (*n =* 100)	22.11	14 (14.14)	6 (6.06)	50 (50.00)	30 (29.29)	1.96
Postpartum (*n =* 180)	22.38	26 (14.44)	3 (2.22)	99 (55.00)	51 (28.33)	1.97
Non-perinatal (*n =* 402)	22.31	63 (15.67)	10 (2.48)	190 (47.26)	139 (34.57)	2.01
HIV Status						
HIV positive (*n =* 89)	21.81	15 (16.85)	3 (3.37)	51 (57.3)	18 (20.22)	1.83
HIV negative (*n =* 421)	21.90	92 (16.10)	19 (3.32)	310 (54.10)	152 (26.53)	1.91
Low birth weight						
Yes (< 2.5 kg) (*n =* 55)	23.5	6 (10.71)	0 (0)	23 (41.70)	27 (48.21)	2.25
No (≥ 2.5 kg) (*n =* 131)	22.1	30 (22.90)	2 (1.53)	65 (49.62)	34 (25.95)	1.78

### EPDS item-10 scores

**Figure 1:**
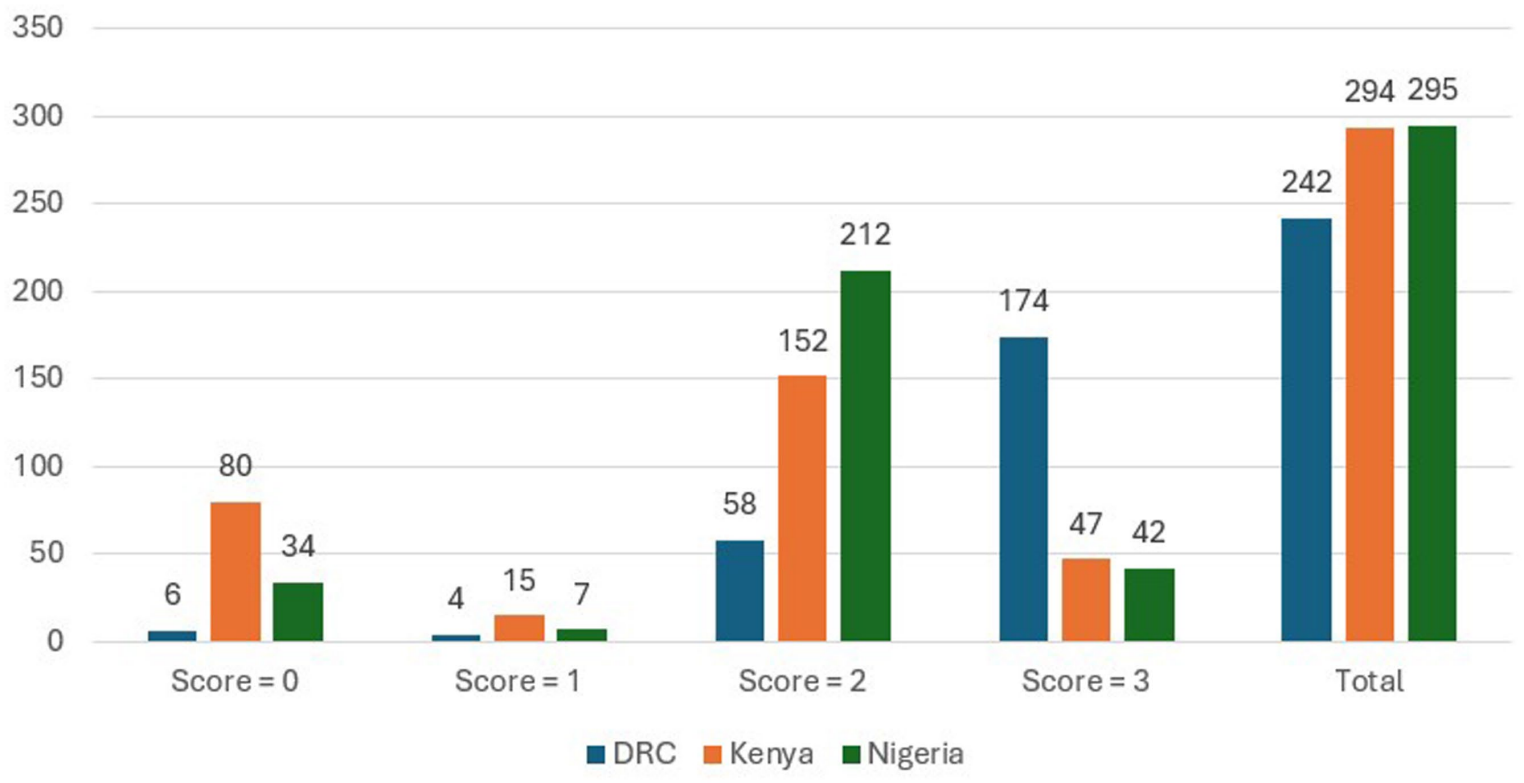
EPDS item-10 scores by country (“The thought of harming myself has occurred to me”) score categories: ‘never’ (score = 0), ‘hardly ever’ (score = 1), ‘sometimes’ (score = 2), and ‘yes, quite often’ (score = 3). Bar graphs and labels depict the number of item-10 responses by score (0–3) and country, and to the far right, the total number of item-10 responses by country irrespective of score (*N =* 831).

Figure 1 illustrates the distribution of item-10 scores by country. Most participants (*n =* 711, 85.6%) responded affirmatively to item-10 (“*The thought of harming myself has occurred to me*”) with “sometimes” (score 2) being the overall mean score and the most common score across countries (46.8% in Kenya, 53.7% in Nigeria, and 51.7% in DRC). The country with the largest proportion of participants responding “yes, quite often” (score 3) to item-10 was Kenya (*n =* 135, 46.1%). In Nigeria and DRC, “sometimes” (score 2) accounted for the largest proportion of respondents (53.7 and 51.7%, respectively). The mean score for item-10 was highest in the DRC at 2.6, while the mean item-10 score in Nigeria was 1.86 and 1.56 in Kenya. Similar to mean EPDS scores, higher mean item-10 scores were noted among women caring for either no children or for >6 children in the household, as well as for women meeting criteria for LBW as compared to those with normal (or higher-than-normal) birth weights. Table 3 details item-10 scores for participants based on various sociodemographic characteristics.

### Correlates of suicidal thoughts and contemplation

Related to their responses to item-10 of the EPDS, 125 participants (15%) offered explanatory comments that provide insight into the circumstances and contexts surrounding their thoughts of suicide. Participants identified multiple interrelated socioecological factors as correlates of suicide contemplation (Table 4). Identified by 47 of the 125 women (37.6%), the topmost cited trigger of suicidal thoughts (or reported suicidal attempts) was the general feeling of being *“tired of this life,” “tired of this work,”* or because of *“the hardship,” “the suffer is too much,”* and “*nothing goes good for me.”* Other comments referenced a lack of food for self/children (*n =* 33, 26.4%), a lack of money/work to school/care for self/children (*n =* 27, 21.6%), traumatic experiences of abuse, exploitation, and violence (*n =* 20, 16.0%), poor living conditions or lack of shelter (living outdoors) for self/children (*n =* 19, 15.2%), and lack of social support (*n =* 12, 9.6%).

**Table 4:** Text analysis of contextual data related to thoughts of self-harm or suicide.

Frequency (*N =* 125)	Correlates of suicidal thoughts and contemplation (factors/concepts)	Overarching themes
47 (37.6%)	Tired of “this life,” tired of “this work,” tired of hardship and suffering (despair)	Desperation
33 (26.4%)	Lack of food for self/children	Deprivation
27 (21.6%)	Lack of money/work to school/care for self/children	Deprivation
20 (16.0%)	Traumatic experiences of abuse, exploitation, or violence	Desperation; Traumatization
19 (15.2%)	Poor living conditions or lack of shelter (outdoor sleeping)	Deprivation
12 (9.6%)	Lack of social support	Isolation
12 (9.6%)	Societal stigma	Marginalization
11 (8.8%)	Illness or injury of a child	Desperation; Traumatization
11 (8.8%)	Loss or death of child(ren) or death of a loved one	Desperation; Traumatization
10 (8.0%)	Abandonment by, separation from, or lack of partner/father of child(ren)	Isolation
8 (6.4%)	Family rejection	Isolation
8 (6.4%)	Inability to afford hospital or medicine for self/child(ren)	Deprivation
6 (4.8%)	Unwanted or unaffordable pregnancy	Desperation; Deprivation
3 (2.4%)	Living with HIV or other sexually transmitted infection	Marginalization
2 (1.6%)	Arrest or incarceration	Criminalization; Traumatization

Among the 20 participants who cited traumatic experiences as triggering their suicidal thoughts, 10 (50%) specifically reported *“rape”* or *“gang rape”* as the inciting traumatic event. One of these cases involved a 15-man gang rape and in another case, a Congolese MFSW with an EPDS item-10 score of 3 described, *“police officers who raped me one after another while I’m 5 months pregnant.”* Similarly, police corruption and impunity were emphasized by another MFSW from DRC (item-10 score of 3) who stated, *“that idea [of suicide] came in my head when I got raped by policers, now I’m pregnant and I do not know what to do.”* One MFSW from the DRC with an item-10 score of 3 stated, *“once I tried to kill myself; I was raped by five men because I sleep on tables outside,”* highlighting the interactions between two or more socioecological correlates of suicidal contemplation—in this case, experiences of violence and poor living conditions.

Notably, of the 125 participants, 19 (15.2%) reported failed suicide attempts in the past. At an extreme, one MFSW admitted to a total of six previous suicide attempts, *“I tried to kill myself by trying to drown in the lake four times. Twice I tried to hang myself”* (DRC, item-10 score of 3). Protective factors were infrequently mentioned by participants; however, these included their children who would be left alone and for whom they wanted to continue living (*n =* 4, 3.2%), their faith (*n =* 2, 1.6%), and substance use as a distraction or coping mechanism (*n =* 1, 0.8%). One Congolese MFSW with an item-10 score of 2 commented, “*Sometimes the idea of committing suicide comes to me, but when I think of my children, I give [it] up*.”

## Discussion

The current study demonstrates the utility of the EPDS screening tool for the detection of *probable* depression and perinatal depression in MFSW communities. Previous studies have demonstrated the utility of 9-item Patient Health Questionnaire (PHQ) (23), the 2-item PHQ (24), the 15-min Mini International Neuropsychiatric Interview (25), and the 20-item Centre for Epidemiological Studies Depression Scale (26, 27) to screen or diagnose depression in FSW populations. The current study represents the first study to employ the EPDS screening tool for depression among perinatal and non-perinatal MFSW populations. Additionally, the exploratory thematic analysis of contextual narrative data offers salient insights into factors associated with suicidal thoughts in this particular study group of marginalized women.

Although the extant literature suggests that the EPDS cut-off score of 10 or 11 offers maximal sensitivity, specificity, and predictive value, higher cut-off scores improve specificity though at the expense of sensitivity (34, 35, 41). Despite applying the more specific, less sensitive higher cut-off score of ≥ 14 in the current study, only 29 (3.5%) participants scored 13 or less (data not shown), with an average lower EPDS score of 11, indicative of *possible* depression. Interestingly, 19 (65.5%) of the 29 MFSW scoring under this cut-off threshold were Nigerian accounting for 6.4% of the Nigeria cohort. Among the other 10 participants, 7 were Congolese (2.9% of the DRC cohort) and 3 were Kenyan (1.0% of the Kenya cohort). The remainder of the sample (*n =* 802, 96.5%) scored ≥ 14 on the EPDS, indicating *probable* depression, although the mean EPDS scores in all three countries fall within the range of 19–30 which corresponds to “severe depression” on more conventional scales of depression severity (51).

As a validated screener and reliable proxy with high specificity for clinical depression, these EPDS scores indicate “probable depression” impacts 96.5% of the pooled sampled population. MFSW “probable depression” percentages of 99, 97.1, and 93.6% in Kenya, DRC, and Nigeria, respectively, are significantly higher than the latest WHO Global Health Observatory data which estimate the general population prevalence of depression in these three LMIC at <5% of the population (4.4% Kenya, 3.9% DRC, and 3.9% Nigeria) with a slightly higher incidence among females compared to males (52–54). Except for a depression prevalence of 100% noted among cisgender and transgender FSWs living with HIV in the Dominican Republic (55), the sample-based prevalence of 96.5% in this multi-country study of MFSW is one of the highest reported in the literature among the already unusually high levels of depression in FSW in LMICs (e.g., 52.7% in South Africa, 82% in Nepal, 86% in Mexico) (23–28). While it is invariably possible that such a large proportion of the study sample would satisfy clinical diagnostic criteria for depression if tested, especially in the wake of the COVID-19 pandemic, further research is needed to determine the optimal cut-off score and confirm the EPDS scores corresponding to depression severity levels on more conventional scales. Likewise, even with the use of higher cut-off scores, the EPDS may be unable to achieve specificity levels high enough to reliably predict “probable depression” among highly stigmatized, traumatized, and socioeconomically marginalized populations of women. Such shortcomings of the EPDS may reflect the internal resilience and coping strategies built across a lifespan of chronic trauma.

Particularly concerning is the elevated risk of suicidal ideation, suicide attempt, and completed suicide associated with depressive disorders (29). Such alarming levels of depression call for emergent deployment of trauma-informed, culturally-sensitive resources to address the mental health needs of this HtR vulnerable population of women. Arguably, however, mental health services will remain inaccessible to FSW without first overcoming the stigma associated with sex work and mental illness. Thus, urgent investigations of alternative models of care delivery are needed to guide targeted interventions and ensure high penetration rates. Some potential options for integrated care include collocating mental health care with services sought out by FSW such as those offered by SWOs as well as NGOs established in FSW communities and providing HIV care, reproductive health, and maternal child health care services. Education and training of SWO workers may allow for expansion of mental health first aid programs and peer support networks through community care models (e.g., peer support specialists, peer support groups, doulas) among FSW living in community. Integrating community and person-centered care models into SWO/NGO efforts as an adjunct to clinical care may buffer the harms of stigma on help-seeking intentions. Community care models can amplify protective factors (i.e., children to live for) and potentially reduce the risk of suicidal ideation. Strength-based therapy and faith-based counseling are potential therapeutic approaches that could be studied and leveraged by SWO/NGO to provide integrated services to FSW and MFSW that foster internal empowerment, enhance resourcefulness, and reframe challenges.

While mental health resources are critical for treatment, targeted interventions that address the socioecological factors underpinning the high levels of depression and suicidal contemplation among MFSW in LMIC must be part of a broader, comprehensive solution. Although the EPDS was not designed to estimate the prevalence of suicidal ideation or predict suicide risk (no assessment of intent or plan), item-10 scores and the corresponding narrative explanations volunteered by participants offer key insights into the circumstances and context surrounding thoughts of suicide in MFSW (Table 4). Corroborating the literature on the lived experiences of FSW in LMIC (17, 19, 21, 56–58), the correlates of suicide contemplation identified by women in this study centered around themes of desperation (hopelessness), deprivation, isolation, marginalization, criminalization, and traumatization, highlighting the need for policies and programs that prevent and ameliorate the social, economic, and environmental contexts of this marginalized group of women and their children. Based on the study’s exploratory findings, financial investments and interventions focused on the accessibility, affordability, and acceptability of health care services for MFSW as well as the availability of social assistance programs for food, safe shelter, childcare, and early school age education, could have a significant beneficial impact on the quality of life and the physical and mental health of FSW mothers and their children.

Furthermore, the profound social isolation and marginalization experienced by MFSW—on the basis of their identity as women who transgress societal, sexual, and gender norms—forces these women to operate on the margins of society in psychosocially desperate conditions and subject to financial destitution, exploitation, and violence, often with no legal or safe recourse from the abuse and trauma. The devastating impacts of this lived experience on the physical and mental health and wellbeing of MFSW and their children must be prioritized by state governments and institutions. Potential interventions worth investigating include anti-stigma public campaigns that challenge and transform cultural and societal attitudes that undergird discrimination against FSW. In addition, the development and strengthening of laws and policies that protect FSW from violence and police corruption with impunity must be prioritized and enforced. Efforts to improve mental health outcomes in MFSW and their children must address these socioecological injustices, combat gender inequities that disproportionately impoverish women and their children, and protect the health and human rights of women and children.

Finally, data from the sampled population in this study suggest that MFSW in the DRC, younger age (18–24 years), number of children in the household at the extremes (0 and > 6 children), and LBW status in a recent pregnancy might be associated with higher EPDS and item-10 scores. Although more robust investigation would be needed to confirm or refute statistically significant differences, the current findings could be related to possibly more challenging sociopolitical circumstances in the DRC, younger age entry into sex work, reduced coping abilities in younger years, death and loss of children at one extreme and caregiver strain with larger numbers of children at the other, and the untoward effects of depression on birth weight. Although the economic strain of caregiving for multiple dependent children (some of whom may be the informally “adopted” children of deceased MFSW peers), pregnancy and postpartum periods, positive HIV status, and low birth weights have all been associated with depression (5, 28, 32, 55, 59), further research is needed to determine if any apparent differences in the mean EPDS scores and mean item-10 scores noted in this sample based on these factors are statistically significant. The absence of any statistical significance, however, may simply reflect the design of the EPDS as a screening tool rather than a dose-dependent measurement of incremental severity. Nonetheless, the utility of EPDS for detecting depression in marginalized hard-to-reach populations is directly related to its sensitivity and specificity profile, brevity, and low-cost application for rapidly identifying women, regardless of perinatal status, who may benefit from referral to mental health services.

### Limitations and strengths

Although the current study employs a well-studied and validated 10-item screening tool for depression, it is possible that MFSW responses were subject to social desirability bias, leading to attenuated severity in the responses and lower scoring, particularly for item 10 which addresses a particularly stigmatizing aspect of mental health. Additionally, selection bias due to the study’s non-random sampling method and potential underlying differences between MFSW who opt to enroll in the study versus those who decline cannot be excluded. This study did not collect any information on MFSW who declined to participate, inclusive of reasons for non-participation. Finally, the use of convenience sampling, while often necessary for gaining access to and studying HtR populations like marginalized FSW in LMICs, is a notable limitation to the generalizability of the findings and precludes an analysis of statistical differences across groups.

Nevertheless, critical access to this HtR vulnerable group of women was achieved with the assistance of local partners staff on the ground who are trusted within the FSW community and long-time collaborators with the research team’s non-profit organization in the delivery of services to these women and their children. Building relationships with a broader swath of the FSW population may, over time, allow for random sampling in future research that would lend itself to statistical comparisons across groups. Finally, the examination of the correlates of suicide contemplation was exploratory and allowed for the inductive identification of themes without assumptions or priming questions. Future studies are needed to expand on protective factors.

## Conclusion

Depression is associated with detrimental behaviors such as substance use, self-harm, and suicide. Perinatal depression is particularly insidious as it carries the potential for significant negative outcomes for the mother, child, and maternal–infant bond. The current study demonstrates the utility of employing the Edinburg Postnatal Depression Scale to screen for *probable* depression and estimate its prevalence among perinatal and non-perinatal MFSW in three sub-Saharan LMIC. Within the study sample, the prevalence of *probable* depression was alarmingly high, and the socioecological factors identified by MFSW as associated with their thoughts of suicide bear themes of desperation (hopelessness), deprivation, isolation, marginalization, criminalization, and traumatization. Effective targeted interventions require the investment of resources into mental health screening and treatment services that meet the needs of MFSW, as well as the development of policies and programs that address the socioecological injustices—such as chronic food insecurity, harsh living conditions, heightened experiences of violence, and inability to provide for the needs of their children—underpinning the alarmingly high prevalence of *probable* depression among populations of MFSW in LMIC.

## Data Availability

Deidentified aggregate data used for this analysis is presented in the article. Additional inquiries can be directed to the corresponding author, BW, who will consider requests for the purpose of a research partnership or provision of services to female sex workers and their children.
